# Multimodal Data Fusion for Whole-Slide Histopathology Image Classification

**DOI:** 10.1007/s41666-025-00212-w

**Published:** 2025-09-03

**Authors:** Yiran Song, Mousumi Roy, Minghao Zhong, Liam Chen, Mingquan Lin, Rui Zhang

**Affiliations:** 1 Division of Computational Health Sciences, Department of Surgery, University of Minnesota, Minneapolis, MN USA; 2 Department of Laboratory Medicine and Pathology, University of Minnesota, Minneapolis, MN USA

**Keywords:** Multimodal learning, Whole-slide image, Pathology, Clinical report, Classification

## Abstract

Whole slide images (WSIs) are critical for cancer diagnosis but pose computational challenges due to their gigapixel resolution. While automated AI tools can accelerate diagnostic workflows, they often rely on precise annotations and require substantial training data. Integrating multimodal data—such as WSIs and corresponding pathology reports—offers a promising solution to improve classification accuracy and reduce diagnostic variability. In this study, we introduce MPath-Net, an end-to-end multimodal framework that combines WSIs and pathology reports for enhanced cancer subtype classification. Using the TCGA dataset (1684 cases: 916 kidney, 768 lung), we applied multiple-instance learning (MIL) for WSI feature extraction and Sentence-BERT for report encoding, followed by joint fine-tuning for tumor classification. MPath-Net achieved 94.65% accuracy, 0.9553 precision, 0.9472 recall, and 0.9473 F1-score, significantly outperforming baseline models (*P* < 0.05). In addition, attention heatmaps provided interpretable tumor tissue localization, demonstrating the clinical utility of our approach. These findings suggest that MPath-Net can support pathologists by improving diagnostic accuracy, reducing inter-reader variability, and advancing precision medicine through multimodal AI integration.

## Introduction

In digital pathology, histological sections are scanned as whole slide images (WSIs), which contain detailed information on tissue and cell morphology, as well as microenvironments. Microscopic analysis of pathology images is the “gold standard” for cancer diagnosis [1]. However, reading WSIs with gigapixel resolution is time-consuming, creating an urgent need for automated computer-assisted diagnosis. AI algorithms can expedite diagnosis, assisting pathologists and saving patients’ time. While computers can accelerate the diagnostic process, the enormous resolution size, approximately 1 gigapixel, makes it impractical to obtain precise and exhaustive annotations for model training. These large (~ 1 GB) images, containing gigapixels of data, pose significant challenges for deep learning pipelines—not because of model design limitations, but due to the substantial computational demands they impose, including memory usage, I/O throughput, and GPU processing capabilities. While parallel training strategies such as all-reduce are available in modern deep learning frameworks (e.g., PyTorch), efficiently distributing and synchronizing the high-volume data required for WSI analysis can still exceed the capacity of many hardware configurations, making full-scale parallelization impractical in routine settings. To address this, smaller patches are extracted from regions of interest (ROIs) and analyzed using 2D convolutional neural networks (CNNs) or traditional feature extraction techniques.

On the other hand, accurate diagnosis is critical for personalized treatment, prognosis, and guiding therapeutic interventions. The goal of the classifier is to identify the class of cancer, i.e., cancer subtype classification so that doctors can provide significant treatment to the patient. Cancer subtype classification models can be used clinically to tailor treatment plans, predict patient prognosis, guide disease monitoring, and select appropriate therapies. By identifying specific cancer subtypes based on molecular and genetic characteristics, these models enable oncologists to choose targeted therapies, thereby improving treatment efficacy and minimizing unnecessary side effects and toxic responses associated with non-specific or overly aggressive treatments. Accurate subtype classification also helps predict recurrence, match patients to clinical trials for experimental treatments, and avoid over-treatment in less aggressive cases. Additionally, the model aids in research by supporting biomarker discovery and understanding drug resistance, ultimately advancing precision medicine in oncology.

Fine-tuning pre-trained models or using multiple-instance learning (MIL) are common approaches, especially when only WSI-level labels are available [2, 3]. ROIs are defined using expert annotations [4], pre-trained segmentation models [3], or image features, and MIL aggregates patch information for supervision [5, 6]. Several MIL-based methods have been developed for WSI classification, including ABMIL [7], ACMIL [6], TransMIL [8], and DSMI [5], each addressing traditional MIL limitations. These methods leverage attention-based pooling, Multiple Branch Attention, Transformer-based frameworks, novel aggregators, self-supervised contrastive learning, and pyramidal fusion to improve WSI classification accuracy and interpretability, with some eliminating the need for localized annotations. While single-modality approaches offer valuable insights, they often fall short in capturing the full complexity of diseases [9]. In clinical practice, physicians often consider information from multiple sources—such as imaging results, clinical and demographic data, and family history—to provide an accurate diagnosis for the patient.

Multi-modality approaches combine the strengths of various data sources, allowing for a more comprehensive assessment of patient health [9]. Computational methods can help mitigate human interpretation inconsistencies and improve algorithm performance by integrating multiple data types. Recent studies have explored pathomic fusion, which combines pathology images, cell graphs, and genomic data to enhance prognosis and diagnosis. For example, Chen et al. [3] introduced a tensor fusion network, while Braman et al. [4] expanded this work to four modalities and introduced an orthogonal loss to ensure feature diversity. Wang et al. [5] utilized the outer product for intermodal and intramodal feature interactions, achieving superior performance compared to simple concatenation and other methods. These approaches demonstrate the potential of multi-modal fusion for effective decision support tools. While predictions based solely on historical WSI data are well established, multi-modal data-driven models remain unexplored in computational pathology. Embracing multi-modality in healthcare is essential for advancing personalized medicine.

WSIs, with their detailed information on tissue morphology, microenvironments, and cell interactions, complement genomic, radiology, and clinical data, making them invaluable for disease diagnosis. Combining WSIs with other data sources like clinical reports, radiology, and genomics can enhance algorithm performance. Non-image modalities such as lab tests, clinical features, and demographic information are vital for diagnosis and prognosis. These modalities include structured and free-text data, requiring specific preprocessing and extraction methods. The success of pre-trained language models (PLMs) in the general domain has led to the development of specialized PLMs in the medical and biomedical fields, which learn language representations useful across tasks without the need for training from scratch. These transformer-based PLMs, trained on large corpora of unlabeled text using self-supervised learning, can be fine-tuned for various downstream tasks by adding task-specific layers [1]. In the evolving field of computational pathology, multi-modal models that combine pathology images with other data sources, such as reports, represent a relatively unexplored yet promising frontier. Unlike traditional methods that rely solely on image classification, multi-modal approaches integrate the strengths of computer vision and natural language processing to create a more comprehensive understanding of the data. This fusion of modalities enables artificial intelligence to process and interpret information in a more human-like manner, enhancing performance across tasks.

For instance, clinical reports contain unstructured text that necessitates natural language processing (NLP) techniques including preprocessing methods such as tokenization, NLP toolkits like ScispaCy [10], and pretrained language models such as BERT [11], to extract meaningful features. Medical language models like ClinicalBERT [12] have also been used for text embeddings and have been compared to BERT for multi-modal prediction tasks [13]. Recent techniques, such as Sun et al.’s [14] deep learning framework for breast cancer prognosis, integrate multimodal data through individual DNNs and a weighted score-fusing module. Therefore, combining WSIs with other data sources such as clinical reports, radiology, and genomics can enhance algorithm performance [15]. Notably, even a small number of clinical features may improve predictions, making data integration a valuable approach [15]. For instance, Yan et al. [16] utilized 29 clinical features, including demographic information (e.g., age, sex), disease history and tumor details—extracted from electronic health records. These clinical features were encoded using a denoising autoencoder, while image features were extracted using a ResNet-18 with average pooling. The final decision combined all features through three fully connected layers. However, prior studies have found that naively integrating WSIs with clinical data—such as through simple feature concatenation—can result in suboptimal performance due to poor cross-modal alignment, feature imbalance, or insufficient modeling of interactions between modalities [1]. To address these limitations, we propose an end-to-end multimodal deep learning framework, MPath-Net, that jointly integrates WSI and text-based features for tumor subtype classification. Prior work has shown that intermediate or late fusion strategies, which combine modality-specific features after initial encoding, are often more effective than early fusion approaches in handling heterogeneous data, especially when modalities differ in scale, structure, or availability [17]. In our framework, we adopt a feature-level fusion strategy—a form of intermediate fusion—in which 512-dimensional image and text embeddings are concatenated and passed through trainable layers that learn cross-modal interactions. This allows the model to jointly reason over both visual and textual signals, improving interpretability and classification performance. While feature-level fusion does not directly reduce intra- or inter-observer variability, it can help mitigate their downstream effects by capturing complementary patterns in both modalities. For instance, textual descriptions may highlight diagnostic context that helps disambiguate subtle or subjective image features. We focus here on demonstrating the utility of multimodal fusion in a weakly supervised setting; exploring comparisons against alternative fusion methods is a promising direction for future work.

Given the large size of WSIs, which are typically processed in smaller patches, we employed a MIL method for image feature extraction. These features, extracted from the second-last layer of the deep neural network (DNN), were then concatenated with text-based embeddings from the second-last layer of a Sentence-BERT transformer model. The combined image and text features were subsequently passed through custom fine-tuning layers to generate the final tumor classification output, demonstrating the potential of multi-modal models to improve disease diagnosis by leveraging complementary data sources. We named our proposed model as MPath-Net (Multimodal Pathology Network). Our research marks a pioneering effort in conducting a comprehensive comparative analysis of cutting-edge state-of-the-art MIL-based methods for WSI classification, specifically targeting multiple cancer subtype classification for two primary sites: kidney and lung, utilizing data from the GDC data portal. Moreover, we have developed an innovative end-to-end multi-modal deep learning pipeline, a first in the pathology domain, which integrates WSI image-based and text-based features to create a robust multimodal model MPath-Net for cancer subtype classification. Notably, our approach employs an end-to-end training process, in which the image classifier and downstream fusion layers are trained jointly, while the transformer-based text encoder is kept frozen to preserve pretrained contextual representations. The image encoder is initialized using weights from self-supervised pretraining but remains fully trainable during multimodal optimization. This design enables the model to learn synergistic representations across modalities and achieve more effective integration of image and text features. We believe this framework offers a robust and reproducible strategy for advancing cancer diagnosis and subtype classification.

## Materials and Methods

### Data

In this study, we use the Cancer Genome Atlas (TCGA) dataset [18] to evaluate the proposed method. TCGA is a cancer genomics program comprising around 20,000 primary cancers and matched normal samples across 33 cancer types [18], including 10 rare cancers. A collaboration between NCI and NHGRI, TCGA aims to create comprehensive genomic maps of major cancer types and subtypes. Analyzing matched tumor and normal tissues from 11,000 patients, the TCGA data are hosted at the NCI GDC [19], including tissue slides and reports for various cancer types. All participants provided informed consent at the time of original study entry. Human Ethics and Consent to Participate declarations: not applicable. This study used publicly available, de-identified data and does not constitute human subjects research.

**Fig. 1:**
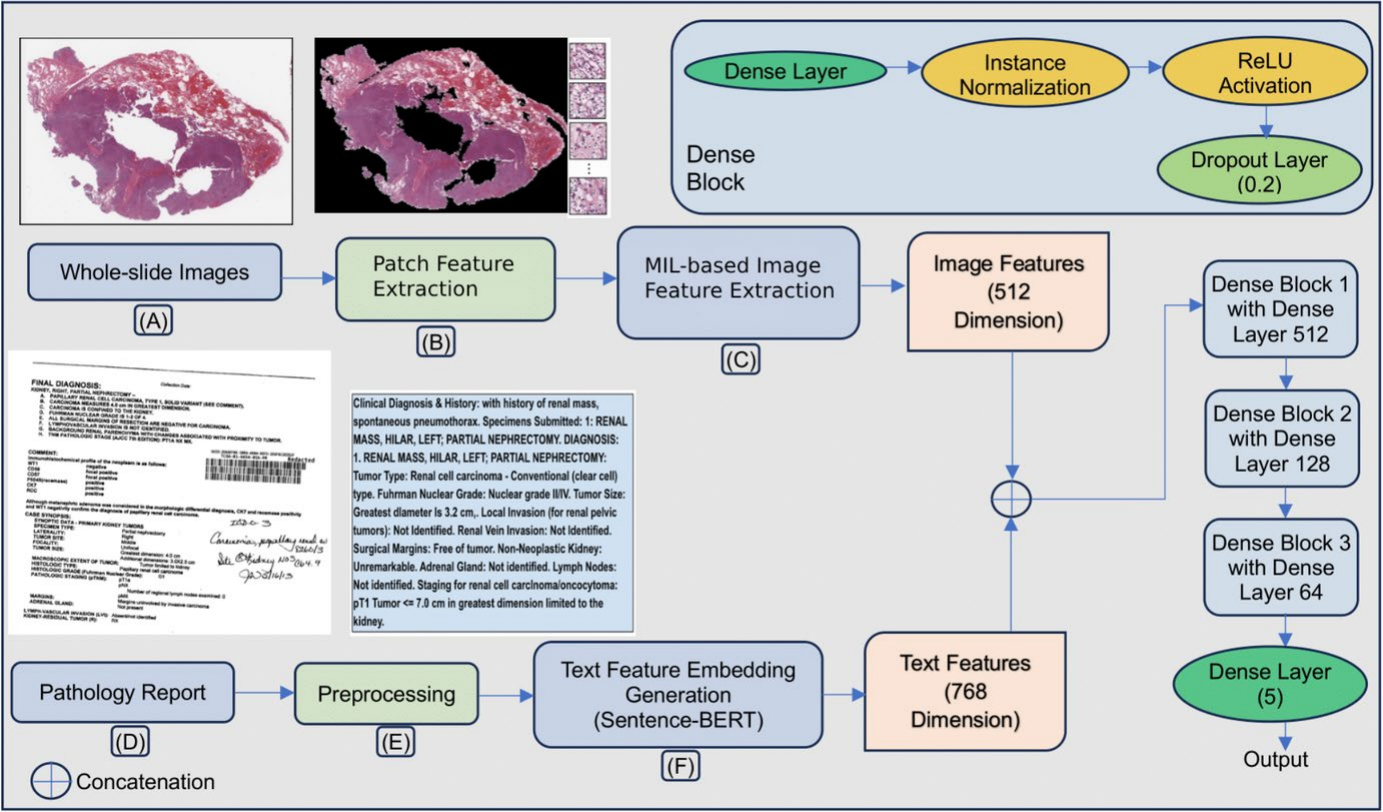
Block diagram of our proposed end-to-end multi-modal WSI classification model MPath-Net, (**A**) A typical WSI; (**B**) Patch extraction from WSI; (**C**) Image feature extraction; (**D**) A typical pathology report in pdf format; (**E**) Preprocessing of pathology report; (**F**) Text feature embedding generation using Sentence-BERT model

To extract this data, we used the GDC Data Transfer Tool [18]. The GDC Data Model organizes data as a Directed Acyclic Graph (DAG) with interconnected entities characterized by detailed attributes. The GDC offers robust filtering for users to refine searches [18] and download files individually or in bulk using the GDC Data Transfer Tool. This tool, a standalone application, simplifies large-scale data downloads, supported by a manifest file listing all files. Figure 1(A) showcases a representative pathology image from the dataset. The size of each such WSI is ~ 1 GB.

We selected the kidney and lung as the primary sites with a total of 5 different disease types. The reason behind choosing these two primary sites is that they are two major regions consisting of many example cases (i.e., rich in data points) with tumor subtype variations. A total of 1684 data points from the records are spanned through the kidney and lungs with a distribution of 916 and 768 records, respectively. Additionally, we chose five specific disease types associated with these organs, including “Kidney Renal Clear Cell Carcinoma,” “Kidney Renal Papillary Cell Carcinoma,” “Kidney Chromophobe,” “Lung Adenocarcinoma,” and “Lung Squamous Cell Carcinoma,” respectively. When selecting medical reports, we prioritize report quality, only considering PDF reports which were clear and readable, and discarding the rest. The number of samples used in our experiments, categorized by primary site and disease type is presented in Table 1. We randomly split the dataset into training (70%), validation (10%), and testing (20%) sets at the patient level. This fixed split was used for all models to ensure consistent evaluation.

**Table 1:** The number of samples categorized by primary site and disease type

Primary site	Disease type	No. of samples
Kidney	Kidney Renal Clear Cell Carcinoma (KIRC)	524
	Kidney Renal Papillary Cell Carcinoma (KIRP)	280
	Kidney Chromophobe (KICH)	112
Lung	Lung Adenocarcinoma (LUAD)	488
	Lung Squamous Cell Carcinoma (LUSC)	280

### Proposed Framework

This study aims to develop an integrated classification system that combines WSI and pathology reports to enhance image classification accuracy in cancer diagnosis. Specifically, we developed an end-to-end multimodal deep learning framework MPath-Net as depicted in Fig. 1. First, WSIs are preprocessed into smaller patches and passed through a Dual-Stream Multiple Instance Learning (DSMIL) network [5] for image feature extraction. The DSMIL model includes a ResNet-18 [20] backbone initialized with weights released by the original DSMIL authors (pretrained using self-supervised contrastive learning on TCGA WSI patches). In our framework, we retain the DSMIL architecture, remove its final classification layer, and extract 512-dimensional feature vectors from the output of the MIL aggregator, which is trained on our classification task. Simultaneously, raw pathology reports are embedded into 768-dimensional vectors using a pretrained Sentence-BERT model. To reduce the modality gap and match feature dimensions, we use a trainable multi-layer perceptron (MLP) to project the text embeddings to 512 dimensions. These image and text features are then concatenated into a 1024-dimensional vector and passed through fully connected layers—beginning with a 512-neuron layer—following conventions in MIL-based classification. During training, the image encoder (ResNet-18 and MIL aggregator), fusion MLP, and classifier are trainable, while the Sentence-BERT model remains frozen. This setup allows us to fine-tune the visual pathway and fusion layers while preserving the robust contextual representations from the pretrained language model. Each component of our framework is described in the following subsections. MPath-Net provides a cost-effective and flexible approach to enhance diagnostic accuracy by leveraging complementary information from histopathology images and clinical narratives.

#### Feature Extraction through Histopathology Image Classification Model

WSIs were tiled into fixed-size patches (256 $\times$ 256 pixels). To eliminate background regions and retain tissue-containing patches, an edge-based filtering criterion was applied. Specifically, each tile was processed using the FIND_EDGES operator from the Python Imaging Library to compute edge intensity. The resulting edge map was summarized by the mean pixel intensity normalized by tile area. Patches with low normalized edge scores were discarded, ensuring that only patches with sufficient structural content (e.g., nuclei, ducts) were retained for downstream analysis.

For image feature extraction, we adopted the DSMIL framework proposed by Li et al. [5], which integrates a standard MIL formulation with both instance-level and bag-level classifiers. This dual-stream design enables the model to capture discriminative patch-level features while also reasoning over the entire slide for final classification. We selected DSMIL due to its strong performance on weakly supervised WSI classification tasks, its ability to handle large, variable-size bags, and its interpretability through attention-based scoring.

Following the original DSMIL framework, we adopted the full image processing pipeline, including the ResNet-18 encoder and the MIL aggregator. Specifically, we used the pretrained weights released by DSMIL, which were originally obtained through self-supervised contrastive learning. In our study, we did not perform any additional pretraining or fine-tuning. We removed the final classification layer from the original DSMIL architecture and used the output of the MIL aggregator as the 512-dimensional feature embedding for each slide (Fig. 1(C)). These embeddings were then used in our downstream multimodal fusion module.

In MIL, a “bag” of training samples comprises multiple instances. Each bag is labeled positive if at least one instance is positive, and negative if all are negative, with individual instance labels unknown. For multi-class classification, the bag label is the most frequent class. The bag probability *θ*(*B*) must be permutation-invariant, achieved through instance embedding$f$, aggregation function σ (MIL pooling), and transformation $g:\theta(B)=g(\sigma(f(p_1),...,f(p_n)))$. The functions $f$ and $g$ can be represented by neural networks, making the MIL model highly flexible and adaptable. This approach enables end-to-end training using backpropagation, allowing the model to learn complex relationships between instances and bags. The MIL pooling operation must be differentiable for gradient-based optimization. There are two approaches: Instance-based, where $f$ scores instances and $g$ pools scores, and Embedding-based, where $f$ extracts features, $g$ aggregates embeddings, and produces a bag score. The latter approach achieves better accuracy but makes it harder to identify key instances driving the classifier’s decision [5].

#### Language Embedding Generation using Unstructured Pathology Report Classification

Pathology reports contain rich information requiring deep analysis [21]. We also leverage unstructured pathology reports from TCGA for our experiments. These reports are a valuable yet underutilized resource in cancer research, offering rich diagnostic insights from pathologists. However, the development of report-based models has been limited by the lack of publicly available machine-readable datasets. Recent advances in optical character recognition (OCR) and natural language processing (NLP) techniques have begun to address this gap. As part of our preprocessing pipeline (Fig. 1(D)–(E)), we used Amazon Web Services (AWS) OCR tools to convert 1949 pathology report PDFs from TCGA into editable text, generating a machine-readable corpus;[3] during this process, we removed barcodes, redaction bars, tables, selection elements, handwritten lines, and multiple-choice forms to produce clean, high-quality text files for downstream analysis.

BERT has been adapted for biomedical and clinical applications through variants such as BioBERT [22], Clinical BioBERT [23], and ClinicalBERT [12], which were pre-trained on domain-specific corpora and fine-tuned for downstream tasks. These models use techniques Like WordPiece Tokenization and learn contextual embeddings that enable semantically meaningful sentence representations for classification and other NLP applications. Additionally, PathologyBERT, trained on 347,173 histopathology reports, has demonstrated strong performance in natural language understanding (NLU) and breast cancer diagnosis tasks [24]. In our study, we evaluated five transformer-based language models for text feature extraction: BioBERT, Clinical BioBERT, ClinicalBERT, PathologyBERT [24], and Sentence-BERT [17]. Using a transfer learning approach, we extracted feature representations from each model by taking the output of the penultimate layer, just before the final fully connected layer (Fig. 1(F)). Among these, the multimodal model using Sentence-BERT embeddings achieved the best classification performance and was adopted for our final model variant.

#### Combined Fine-Tune Layers for Final WSI Classification

This section details the experimental setup for our combined analysis. First, each WSI vector from the bag prediction and its corresponding report text embedding vector are input to the DNN model. These features are concatenated across the 3rd axis and passed to the dense layer. Next, the features undergo instance normalization, standardizing inputs to stabilize learning and reduce training epochs. The normalized features are then processed through a series of fully connected layers (512, 128 and 64 neurons) followed by RELU activation and dropout layers with dropout rates of 0.2 each. For this multiclass classification problem with five classes, the final layer is a dense layer with five neurons and a “softmax” activation function. The overall architecture is depicted in Fig. 1. The end-to-end combined model is trained using Adam optimizer [25] with a learning rate of 0.0001 and uses “categorical cross-entropy” as the loss function. This combined model differs from those used to classify images and text separately.

### Experiment Setup and Evaluation Metrics

The classification model’s performance is evaluated using four key metrics: precision, recall, F1-score, and accuracy. Precision measures the proportion of true positives among all positive predictions, recall measures true positives among all actual positive instances, and the F1-score is a harmonic mean of precision and recall. Accuracy measures the proportion of correctly predicted observations among all observations, providing a comprehensive understanding of the model’s performance. The formulas for these metrics are as follows:

$$\mathrm{Precision}\:=\:(\mathrm{TP})/(\mathrm{TP}\:+\:\mathrm{FP})$$

$$\mathrm{Recall}\:=\:(\mathrm{TP})/(\mathrm{TP}\:+\:\mathrm{FN})$$

$$\mathrm F1-\mathrm{score}\:=\:(2\;\ast\;\mathrm{Precision}\;\ast\;\mathrm{Recall})/(\mathrm{Precision}\:+\:\mathrm{Recall})$$

$$\mathrm{Accuracy}\:=\:(\mathrm{TP}\:+\:\mathrm{TN})/(\mathrm{TP}\:+\:\mathrm{FP}\:+\:\mathrm{TN}\:+\:\mathrm{FN})$$

where TP, FP, TN, and FN stand for true positive, false positive, true negative, and false negative respectively.

To estimate 95% confidence intervals for each evaluation metric, we used bootstrap resampling with 200 iterations. In each iteration, $n$ patients were sampled with replacement from the test set (of size $n$), and the model was evaluated on the resulting sample. The 95% CI was computed as the interval between the 2.5th and 97.5th percentiles of the resulting distribution. This procedure was applied consistently across all experiments, and we report confidence intervals in all results tables where applicable.

### Training and Implementation

Model training was conducted on 4-core A100 GPUs. Following the DSMIL framework, we utilized the provided pre-trained weights for patch-level feature extraction, with ResNet-18 serving as the CNN backbone for both the MIL model and SimCLR [26]. For text feature extraction, we employed multiple BERT-based pre-trained transformer models, aligned with our proposed MPath-Net variants (V1–V4). During the end-to-end training phase, we used the Adam optimizer [25] with a fixed learning rate of 0.0001 and a batch size of 1. We evaluate the performance of our proposed model MPath-Net against several state-of-the-art MIL methods, including TransMIL [8], ACMIL [6], ABMIL [7], MaxMIL [6], and MeanMI [6], for WSI classification. To ensure a consistent basis for comparison, all unimodal MIL models in our benchmark experiments were trained using the same SIMCLR-pretrained ResNet-18 encoder as used in MPath-Net. This allows us to isolate the effect of multimodal fusion and architectural differences without introducing confounding factors related to backbone variability. Based on the different BERT-based pre-trained transformer models for text feature extraction, we have several model variations of MPath-Net (V1 to V4).

## Results

One of our model variations MPath-Net_V1, based on different transformer models for text feature extraction and baseline models, is detailed in Table 2. Specifically, we reported the 95% CI values for each metric test accuracy, precision, recall, F1-score and AUC respectively. Notably, the model with SentenceBERT (MPath-Net_V1) achieved an accuracy of 0.9487 (0.9443, 0.9531), the highest precision of 0.9495 (0.9441, 0.9548), the highest recall of 0.9445 (0.9396, 0.9495) and the highest F1-score of 0.9460 (0.9406, 0.9514) whereas the best baseline model ACMIL achieved an accuracy of 0.929 (0.9163, 0.9295), precision of 0.9192 (0.9120, 0.9264), recall of 0.9231 (0.9162, 0.9300) and F1-score of 0.9165 (0.9094, 0.9236), respectively.

**Table 2:** Results on the TCGA dataset with mean value and 95% CI for several baseline MIL models along with our proposed multi-modal model. Bold values indicate the best performance across all methods for each evaluation metric

Method	Accuracy	Precision	Recall	F1-score	AUC
TransMIL	0.9215 (0.9154, 0.9276)	0.9099 (0.9021, 0.9177)	0.9158 (0.9096, 0.9221)	0.9102 (0.9029, 0.9174)	0.9863 (0.9822, 0.9904)
ACMIL	0.9229 (0.9163, 0.9295)	0.9192 (0.9120, 0.9264)	0.9231 (0.9162, 0.9300)	0.9165 (0.9094, 0.9236)	0.9885 (0.9845, 0.9925)
ABMIL	0.9034 (0.8956, 0.9113)	0.9048 (0.8967, 0.9129)	0.8998 (0.8918, 0.9079)	0.8982 (0.8906, 0.9059)	0.9900 (0.9860, 0.9939)
MaxMIL	0.8866 (0.8785, 0.8948)	0.9266 (0.9212, 0.9319)	0.8892 (0.8801, 0.8982)	0.8990 (0.8917, 0.9063)	0.9907 (0.9867, 0.9947)
MeanMIL	0.8248 (0.8152, 0.8345)	0.8595 (0.8508, 0.8681)	0.8279 (0.8185, 0.8373)	0.8266 (0.8167, 0.8365)	0.9810 (0.9768, 0.9851)
DSMIL	0.9274 (0.9211, 0.9348)	0.9163 (0.9089, 0.9247)	0.9245 (0.9135, 0.9327)	0.9135 (0.9004, 0.9216)	0.9875 (0.9748, 0.9927)
MPath-Net_V1(SentenceBERT)	**0.9487** **(0.9443, 0.9531)**	**0.9495** **(0.9441, 0.9548)**	**0.9445** **(0.9396, 0.9495)**	**0.9460** **(0.9406, 0.9514)**	**0.9902** **(0.9861, 0.9942)**

We also investigate the impact of different BERT-based pre-trained transformer models for text feature extraction in the proposed model. As shown in Table 3, MPath-Net_V1, outperforms other variations.

**Table 3:** Results on the TCGA dataset for several baseline MIL models along with our proposed multi-modal model MPath-Net_V1. Bold values highlight the best-performing BERT variant (SentenceBERT), serving as a reference for future researchers

Method	Accuracy	Precision	Recall	F1-score	AUC
MPath-Net_V2 (BioBERT)	0.9144 (0.9098, 0.9189)	0.9218 (0.9179, 0.9260)	0.9093 (0.9056, 0.9147)	0.9141(0.9096, 0.9189)	0.9894 (0.9856, 0.9932)
MPath-Net_V3 (ClinicalBERT)	0.9358 (0.9317, 0.9401)	0.9399 (0.9352, 0.9437)	0.9424 (0.9379, 0.9479)	0.9403 (0.9351, 0.09448)	0.9900 (0.9861, 0.9934)
MPath-Net_V4 (ClinicalBioBERT)	0.9144 (0.9102, 0.9189)	0.9328 (0.9276, 0.9372)	0.9132 (0.9075, 0.9184)	0.9215 (0.9162, 0.9259)	0.9893 (0.9852, 0.9928)
MPath-Net_V5 (PathologyBERT)	0.9037 (0.8997, 0.90.86)	0.9131 (0.9089, 0.9168)	0.9113 (0.9076, 0.9156)	0.9102 (0.9048, 0.9154)	0.9882 (0.9847, 0.9923)
MPath-Net_V1 (SentenceBERT)	**0.9487** **(0.9443, 0.9531)**	**0.9495** **(0.9441, 0.9548)**	**0.9445** **(0.9396, 0.9495)**	**0.9460** **(0.9406, 0.9514)**	**0.9902** **(0.9861, 0.9942)**

Class-wise classification accuracy for MPath-Net_V1 and baseline models is reported in Table 4, where MPath-Net_V1 achieves the highest performance in three out of five classes.

**Table 4:** Class-wise comparison metric (accuracy) for baseline and our proposed MPath-Net_V1

Accuracy	TransMIL	ACMIL	ABMIL	MaxMIL	MeanMIL	MPath-Net_V1
Class0 (KIRC)	0.9285 (0.9223, 0.9332)	0.9285 (0.9224, 0.9332)	0.8571 (0.8517, 0.8623)	0.8571 (0.822, 0.8618)	0.7857 (0.7802, 0.7928)	1.0000 (1.0000, 1.0000)
Class1 (KIRP)	0.9148 (0.9097, 0.9203)	0.9361 (0.9324, 0.9427)	0.9148 (0.9101, 0.9198)	0.9361 (0.9302, 0.9423)	0.8085 (0.8031, 0.8131)	0.9069 (0.8945, 0.9143)
Class2 (KICH)	0.8888 (0.8803, 0.8937)	0.9444 (0.9401, 0.9497)	0.9259 (0.9208, 0.9302)	0.8888 (0.8824, 0.8925)	0.8703 (0.8647, 0.8773)	1.0000 (1.0000, 1.0000)
Class3 (LUAD)	0.9591 (0.9527, 0.9636)	0.9183 (0.9134, 0.9226)	0.9387 (0.9337, 0.9425)	0.9795 (0.9735, 0.9827)	0.9795 (0.9731, 0.9827)	0.9583 (0.9513, 0.9648)
Class4 (LUSC)	0.9090 (0.9019, 0.9137)	0.9090 (0.9031, 0.9123)	0.8787 (0.8729, 0.8823)	0.7878 (0.7821, 0.7935)	0.6969 (0.6912, 0.7037)	0.8709 (0.8669, 0.8743)

An analysis of each component separately (Table 5) reveals that the histopathology image-based model outperforms the medical text-based model. Notably, the histopathology image-only model achieves reasonable classification accuracy, underscoring its diagnostic potential. However, the multi-modal model, which integrates both image and text data, outperforms both unimodal approaches across all evaluation metrics, achieving the highest accuracy (0.9487), precision (0.9495), recall (0.9445), F1-score (0.9460), and AUC (0.9902). Interestingly, while the fused model outperforms both unimodal baselines across accuracy, precision, recall, and F1-score, the image-only model achieves a slightly higher AUC (0.9985 vs. 0.9902). This may be attributed to the image-only model’s sharper decision boundary in ranking predictions when solely relying on high-resolution visual cues. In contrast, incorporating textual features—while improving overall classification metrics—can introduce modality noise or redundancy that slightly softens probability separability in ROC space. This trade-off between ranking capability (AUC) and threshold-based classification metrics (e.g., F1-score) is not uncommon in multimodal settings, and suggests that while fusion improves overall predictive performance, unimodal signals may still offer advantages in certain aspects of model calibration or ranking precision.

**Table 5:** Comparative analysis results for unimodal vs MPath-Net_V1. Bold values indicate that multimodal fusion (MPath-Net_V1) yields performance significantly superior to unimodal approaches across all metrics

Metrics	Image only model	Text only model	Fused model (MPath-Net_V1)
Accuracy	0.9229 (0.9163, 0.9295)	0.7914 (0.7856, 0.7995)	**0.9487** **(0.9443, 0.9531)**
Precision	0.9192 (0.9120, 0.9264)	0.7666 (0.7615, 0.7328)	**0.9495** **(0.9441, 0.9548)**
Recall	0.9231 (0.9162, 0.9300)	0.7248 (0.7168, 0.7295)	**0.9445** **(0.9396, 0.9495)**
F1-score	0.9165 (0.9094, 0.9236)	0.7337 (0.7286, 0.7377)	**0.9460** **(0.9406, 0.9514)**
AUC	0.9885 (0.9845, 0.9925)	0.9647 (0.9601, 0.9732)	**0.9902** **(0.9861, 0.9942)**

To further validate the significant improvement of our method compared to the baseline methods, we performed a *p*-value analysis for all metrics, which is reported in Table 6. We found that the *p*-value for comparisons between our model MPath-Net and each baseline model was consistently < 0.05 for test accuracy, precision, recall and F1-score respectively.

**Table 6:** P-value analysis for our model vs each baseline model

Our model MPath-Net VS	Test accuracy	Precision	Recall	F1-score	AUC
TransMIL	4.2257 × e-12	2.6529 × e-15	6.6337 × e-12	5.3081 × e-14	0.1828
ACMIL	4.6054 × e-10	8.3871 × e-11	9.6949 × e-07	2.3139 × e-10	0.5696
ABMIL	9.1914 × e-21	4.9996 × e-18	8.6669 × e-19	3.4938 × e-21	0.9426
MaxMIL	2.7110 × e-33	5.3034 × e-09	2.9405 × e-23	6.4650 × e-22	0.8532
MeanMIL	4.8561 × e-75	4.4277 × e-51	1.8409 × e-69	8.8320 × e-66	0.0017

**Fig. 2:**
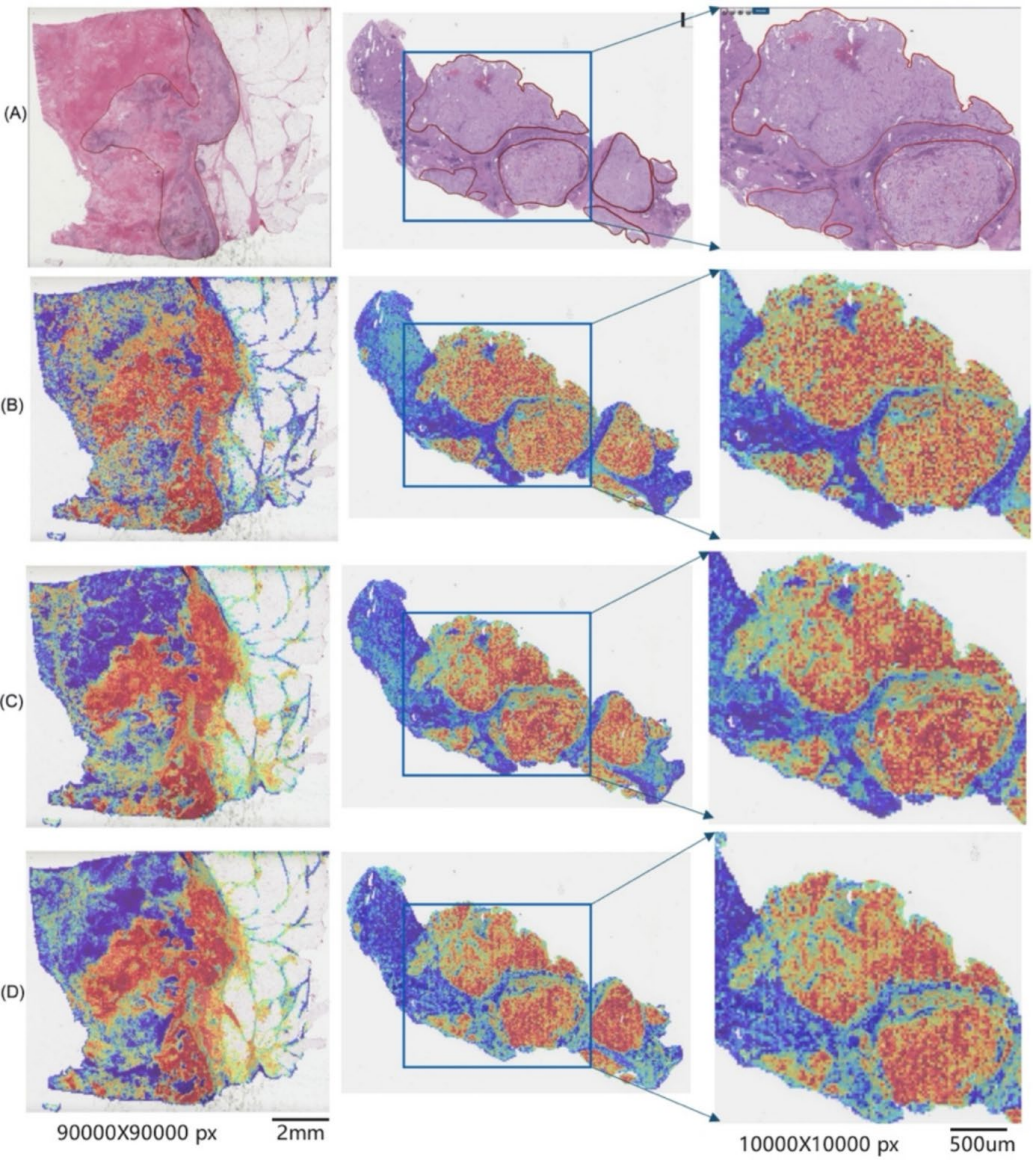
Heatmap visualization of WSI: **A** Original WSI; two baseline methods, **B** ACMIL, **C** ABMIL, and **D** our proposed model MPath-Net. Column 3 shows a zoomed region as marked by a blue box from Column 2

Figure 2 displays visualizations for a selection of WSIs. Attention Heatmaps generated for these WSIs facilitate the identification of tumor tissue as ROIs. The attention scores are normalized between 0 and 1.0, where 0 indicates no evidence of a tumor and 1.0 represents strong evidence of a tumor patch. Utilizing a diverging colormap, the normalized scores are transformed into RGB colors in the slide, enabling effective visualization and interpretability. Notably, regions with high attention are depicted in red in the attention map, while areas with low attention, indicating no evidence of a tumor, are displayed in blue. Specifically, we have demonstrated the attention heatmap for WSIs for ABMIL, ACMIL and our proposed multimodal model MPath-Net_V1 methods.

## Discussion

In digital pathology, despite varied approaches to image analysis, the primary objective is consistent: to detect patterns within the pixels of histopathological images and translate these patterns into clinically meaningful insights. The development of robust image-processing algorithms requires significant effort and a profound understanding of data structure, texture, and variance. While this can be challenging, the ideal solution would be for computers to perform these tasks with a human-like understanding—inferring meaning from images based on examples and experience. Machine learning offers a path to this goal, allowing us to train models that can make insightful predictions on previously unseen data by learning from labeled examples or through pattern discovery without labels.

Traditionally, machine learning in pathology has relied on supervised and unsupervised learning. In supervised learning, models are trained using labeled data with known diagnostic outcomes—much like a pathologist teaching a resident by pointing out key histologic features. In contrast, unsupervised learning identifies inherent patterns in unlabeled data, analogous to allowing trainees to explore slides independently and discover structure on their own.

A widely adopted third strategy in computational pathology is weakly supervised learning, which is particularly well-suited for histopathology datasets where pixel- or patch-level annotations are often unavailable. In our study, we build on this approach by integrating weakly supervised image learning via MIL with textual information from pathology reports. This multimodal fusion enables our model to benefit from both image-level patterns and the diagnostic guidance embedded in clinical text.

Our proposed framework, MPath-Net, combines a MIL-based model for extracting image features with a Sentence-BERT-based model for embedding textual content. These modality-specific features are then fused through fully connected layers to enable joint learning. This is conceptually similar to how pathologists use both slides and clinical notes together to make more informed diagnoses. Our results demonstrate that this multimodal weak supervision approach leads to improved performance over unimodal baselines.

WSIs offer unique, intricate views of tissue morphology, cellular interactions, and the tumor microenvironment, which are often not captured by other data sources like genomic, radiological, or clinical data. This makes WSIs incredibly valuable for deepening our understanding of disease. When these rich imaging data are combined with the structured and narrative information found in pathology reports, it results in a more holistic view of the disease, often enhancing classification accuracy and improving the model’s generalizability. MPath-Net is specifically designed to leverage this combination, seamlessly integrating visual and textual features in an end-to-end manner, unlike many conventional models that freeze layers or reload pre-trained weights. This uninterrupted training process allows MPath-Net to fuse visual and textual information cohesively, which we hypothesize leads to a completer and more accurate model.

The results of our study confirm this hypothesis, with MPath-Net significantly outperforming unimodal models across all evaluated metrics, including accuracy, precision, recall, and F1-score. This performance highlights the advantage of integrating Heterogeneous data sources and demonstrates the synergistic value of combining visual and textual information. In our framework, we adopted a straightforward feature-level fusion strategy: 512-dimensional image features and 512-dimensional text features were concatenated to form a 1024-dimensional joint representation. This combined feature vector was then passed through fully connected layers, enabling the model to learn cross-modal interactions that improve classification accuracy. While more sophisticated fusion methods—such as tensor-based or attention-driven approaches—may further enhance performance, our results show that even simple fusion via concatenation can be highly effective in leveraging complementary multimodal information.

On the imaging side, our model adopts a weakly supervised WSI classification approach, utilizing an MIL-based architecture. Due to the limited tumor region annotations in the GDC TCGA dataset, this method treats each WSI as a collection of smaller image patches, enabling the model to process and analyze the image in parts. By applying self-supervised contrastive learning, MPath-Net is able to extract robust representations for MIL, allowing the model to differentiate between normal and tumor regions without needing exhaustive pixel-level annotations. For the text data, we found that Sentence-BERT outperforms other BERT-based variants in capturing textual nuances, providing more accurate text feature embeddings and providing more discriminative feature embeddings. This may be due to its architecture, which is specifically optimized to produce semantically meaningful sentence-level representations using a Siamese network trained with contrastive objectives. In contrast, other models such as ClinicalBERT and BioBERT are optimized for token-level predictions, which may limit their effectiveness in document-level classification tasks. This sentence-level focus likely made Sentence-BERT more suitable for encoding pathology reports, which consist of structured clinical narratives. To further validate MPath-Net’s interpretability, attention heatmaps generated by the model highlight regions of interest, illustrating the model’s capability to accurately localize tumor regions, which adds a layer of transparency to its decision-making process.

Our statistical analysis further confirms the superiority of MPath-Net, with *p*-values consistently below 0.05 when compared to baseline MIL-based models. This statistical significance indicates a notable improvement over existing methods, underscoring the robustness of our multimodal fusion approach. The model’s ability to consistently perform better than traditional, unimodal approaches highlights the effectiveness of integrating diverse data types and the potential of multimodal learning in digital pathology.

Nevertheless, as with any complex AI model, MPath-Net has its limitations. One current challenge is the limited diversity of datasets; we plan to broaden its application to more heterogeneous data sources, including complex cancer types with varied histopathological characteristics. Additionally, while recent patch-level foundation models (e.g., UNI v2 [27], Virchow 2 [28], GigaPath [29], Hoptimus 1 [30]) offer powerful image representations, our current framework does not incorporate them. Instead, we prioritize accessibility and reproducibility by using a DSMIL encoder trained via self-supervised learning on publicly available TCGA data. This choice ensures transparency and deployability in resource-constrained settings. That said, integrating foundation model features—either by replacing or augmenting our current encoder—represents a promising direction for future work.

Beyond model inputs, we are also exploring enhancements to MPath-Net’s architecture. These include advanced attention mechanisms to better capture intermodal dependencies, alternative word embeddings to enrich textual feature extraction, and improved image compression strategies to maintain data fidelity while reducing computational load. Looking forward, we aim to extend MPath-Net’s functionality to generate automated pathology reports directly from WSIs—a development that could streamline personalized diagnostics and reduce the workload of clinical pathologists by enabling more consistent, efficient reporting for routine and complex cases alike.

## Conclusion

In summary, MPath-Net represents a significant step forward in digital pathology, showcasing the power of multimodal learning by seamlessly integrating WSIs and pathology reports into a unified predictive pipeline. By synthesizing complementary data from multiple sources, MPath-Net not only achieves a high level of classification accuracy of 94.65% but also aligns with the evolving needs of modern, patient-centered diagnostic medicine. This model offers a compelling glimpse into the future of digital pathology, where integrated AI systems can support pathologists in delivering more accurate, data-driven diagnoses with increased efficiency and confidence.

## Data Availability

No datasets were generated or analysed during the current study.
